# Unveiling metagenomic and metabolomic signatures in mild and severe pneumonia caused by Mycoplasma pneumoniae in children

**DOI:** 10.1099/mgen.0.001717

**Published:** 2026-05-20

**Authors:** Yeran Yang, Shitao Lian, Xiao Li, Yu Tang, Yanyan Su, Zeming Zhang, Min Li, Yongli Guo, Zilong He, Yuelin Shen

**Affiliations:** 1 Laboratory for Pediatric Diseases of Otolaryngology, Head and Neck Surgery, MOE Key Laboratory of Major Diseases in Children, Beijing Pediatric Research Institute, Beijing Children’s Hospital, Capital Medical University, National Center for Children’s Health (NCCH), Beijing, 100045, PR China; 2 School of Engineering Medicine, Beijing Advanced Innovation Center for Big Data-Based Precision Medicine, Interdisciplinary Innovation Institute of Medicine and Engineering, Beihang University, Beijing, PR China; 3 Respiratory Department, Children’s Hospital Affiliated to Zhengzhou University, Henan Children’s Hospital, Zhengzhou Children’s Hospital, Zhengzhou, 450018, PR China; 4 Respiratory Department, National Clinical Research Center for Respiratory Diseases, Beijing Children’s Hospital, Capital Medical University, National Center for Children’s Health, Beijing, 100045, PR China

**Keywords:** antibiotic resistance, metabolome, metagenomic sequencing, multi-factor prediction model, *Mycoplasma pneumoniae*

## Abstract

**Background.** *Mycoplasma pneumoniae* (*MP*) is a common causative pathogen of community-acquired pneumonia in children, with clinical presentations ranging in severity. Early stratification and timely intervention are essential for improving patient outcomes. However, a major clinical challenge lies in the limited ability to accurately distinguish between mild and severe cases based solely on early clinical indicators.

**Methods.** This prospective real-world study investigated the differences in microbiome and metabolomics between mild and severe *MP* pneumonia (MPP) in children. Bronchoalveolar lavage fluid samples were collected from 153 children and subjected to metagenomic sequencing and non-targeted metabolomic analysis. Meanwhile, to enhance early diagnostic accuracy, this study developed a machine learning classification model and validated it using a third-party validation set.

**Results.** The results revealed significant alterations in the abundance of specific bacterial communities in the severe group, most notably the coexistence of *MP* and *Alphainfluenzavirus influenzae*, which may contribute to disease exacerbation through synergistic pathogenic mechanisms. Furthermore, the macrolide resistant rate of *MP* in the severe group exceeded 80%, emphasizing the importance of appropriate antibiotic selection. Metabolomic analysis showed a significant enrichment of metabolites related to cellular energy metabolism and immune regulation in severe cases. The model demonstrated exceptional predictive performance, achieving an area under the curve ranging from 0.909 to 0.991, which significantly outperformed conventional clinical stratification methods.

**Conclusions.** These findings elucidate the distinct pathophysiological mechanisms underlying both mild and severe MP infections and provide a promising framework for improving early diagnosis and personalized treatment strategies in paediatric MPP.

**Impact Statement**

- Integrated metagenomic and non-targeted metabolomic analysis of *Mycoplasma pneumoniae* pneumonia (MPP) in children with mild and severe groups.
- Network and correlation analysis of lung microbiota with metabolites and clinical indicators in children with mild and severe groups.
- Multi-factorial early warning model for MPP mild and severe groups was constructed and demonstrated high performance.

## Data Availability

The BioProject number for the raw sequencing data reported in this article is PRJNA1177601. The list of accession numbers refers to Table S3.

## Introduction

*Mycoplasma pneumoniae* (*MP*) is a small prokaryotic micro-organism with a genome of ~820 kb, making it one of the smallest self-replicating bacteria [1]. It has the capacity for self-replication and primarily resides within human bronchial and alveolar epithelial cells [2]. Due to its lack of a cell wall, *MP* is inherently resistant to *β*-lactam antibiotics such as penicillin [3]. *MP* is a common causative agent of community-acquired pneumonia in children, known as *MP* pneumonia (MPP) [4,5]. Following the implementation of non-pharmaceutical interventions for coronavirus disease 2019 (COVID-19) in 2021, the global incidence of MPP declined significantly and remained low throughout 2022 [6,8]. However, since 2023, a sharp resurgence of MPP has been observed in China [9].

The clinical manifestations of MPP vary widely, ranging from mild, self-limiting infections to severe, life-threatening pneumonia. Severe *MP* pneumonia (SMPP) may present with acute respiratory distress syndrome, respiratory failure and long-term pulmonary sequelae such as post-infectious bronchiolitis obliterans (PiBO) and bronchiectasis [10,11]. Moreover, SMPP can lead to extrapulmonary complications involving cardiovascular, neuromuscular, haematological and mucocutaneous systems [12]. Early stratification and timely intervention are critical for improving the outcomes of paediatric MPP. However, current diagnostic strategies remain predominantly dependent on conventional clinical indicators. Due to the prolonged and variable disease course, some patients initially manifest mild symptoms but progress to severe disease within 1–2 weeks after disease onset, consequently missing the critical window for early intervention. Thus, it is imperative to develop more reliable methods for precise and early severity stratification [13].

In recent years, research on *MP* has gained momentum, particularly in the context of post-pandemic surveillance [14]. Molecular epidemiological studies have revealed alarmingly high rates of macrolide resistance in paediatric *MP* infections. In 2022, Xu *et al*. reported a resistance rate of up to 96% in Wuhan between 2020 and 2022 [15]. In 2023, a separate study by Xu *et al*. identified resistance-associated mutations in 71% of 299 *MP*-positive samples from southern China using metagenomic sequencing [16]. That same year, Chen *et al*. documented the dissemination of globally circulating resistant clones within China [17]. In 2024, Li *et al*. analysed over 30,000 PCR test results and 448 metagenomic samples, demonstrating that the resurgence of macrolide-resistant *MP* was driven by the widespread transmission of two dominant epidemic clones. Notably, the EC2 clone harbours the A2063G mutation in the 23S rRNA gene, conferring complete macrolide resistance [18]. Despite these advances, the biological mechanisms underlying the differences between mild and severe MPP remain insufficiently explored. In particular, the microbial and metabolomic distinctions between these two clinical forms – and their potential contributions to disease progression – have not been fully elucidated.

To address these gaps, this study conducted comprehensive microbial and metabolomic analyses of bronchoalveolar lavage fluid (BALF) samples from 153 paediatric patients with MPP, using metagenomic sequencing and untargeted metabolomics. Our objective was to identify key differences in microbial composition and metabolic profiles between mild and severe cases and to develop a machine learning-based diagnostic model that integrates multi-omics data. By improving early diagnostic accuracy, this approach aims to support more effective clinical decision-making and personalized treatment strategies for paediatric *MP* infections.

## Methods

### Study population and sample collection

We conducted a prospective real-world study on 153 children hospitalized for MPP at the Respiratory Department of Children’s Hospital Affiliated to Zhengzhou University, China, between October 2020 and May 2024. MPP was diagnosed based on the following criteria according to the Chinese Pediatric *Mycoplasma pneumoniae* pneumonia Diagnosis and Treatment Guidelines (2023 version) [19]: clinical presentation (fever, cough), chest imaging with infiltrates, a fourfold rise in cold agglutinin titre from the acute to convalescent phase and/or a positive PCR test of sputum/BALF. The exclusion criteria were as follows: disease course of <4 days or >7 days at the time of admission; lack of chest imaging during 4–7 days of disease course; underlying cardiovascular, neuromuscular, haematological or genetic disorders; and congenital airway malformations, chronic lung diseases or asthma.

BALF samples were collected from all enrolled patients on the second day of hospitalization, following standardized clinical procedures to ensure consistency across cases. Sample collection was performed by experienced respiratory clinicians using fibreoptic bronchoscopy under appropriate sedation and monitoring. Immediately after collection, the BALF samples were aliquoted into sterile cryovials to prevent repeated freeze-thaw cycles and were promptly cryopreserved at −80 °C to maintain sample integrity. All procedures adhered to institutional biosafety and ethical standards.

This study was approved by the Ethics Committee of Children’s Hospital Affiliated to Zhengzhou University (Approval No. 2024-KY-0064-001). Written informed consent was obtained from the patients or their legal guardian prior to enrolment.

#### Grouping

All enrolled patients underwent two clinical severity assessments: the first on the day of admission and the second on the seventh day of hospitalization. Based on each assessment, patients were classified into mild MPP (MMPP) and SMPP groups. SMPP is clinically defined as MPP that meets the criteria for severe community-acquired pneumonia according to the Chinese Pediatric *Mycoplasma pneumoniae* pneumonia Diagnosis and Treatment Guidelines (2023 version) and presents with any of the following manifestations: persistent high fever (>39 °C) for ≥5 days or fever for ≥7 days with no downward trend in peak temperature; any of the symptoms of dyspnoea, chest pain or haemoptysis; severe extrapulmonary complications such as neurological complications (including encephalitis, acute disseminated encephalomyelitis and cerebral infarction), cardiovascular complications (including intracardiac thrombus, myocarditis and Kawasaki disease), haematological complications (including immune thrombocytopenia, autoimmune haemolytic anaemia and haemophagocytic lymphohistiocytosis), skin and mucosal complications (including erythema multiforme, Stevens-Johnson syndrome and *MP*-induced rash and mucositis), as well as acute kidney injury, liver failure, acute pancreatitis, rhabdomyolysis, etc.; finger pulse oximetry ≤93% on room air at rest; consolidation area ≥2/3 of a lobe or consolidation involvement ≥2 lobes; and progressive worsening of clinical symptoms, with radiological deterioration more than 50% in 24–48 h.

### Clinical indicators and laboratory testing

In this study, we collated clinical data from 153 Chinese children diagnosed with MPP, including demographic data, clinical presentations, intrapulmonary and extrapulmonary complications, length of hospital stay and the incidence of sequelae such as bronchiectasis and PiBO. The laboratory parameters assessed included inflammation markers and coagulation indicators. Additionally, chest computed tomography (CT) imaging was performed to evaluate the severity of intrapulmonary disease, including lung consolidation, pulmonary necrosis, pleural effusion, pulmonary thrombosis and other pulmonary complications. Bronchoscopies were performed to assess airway conditions, such as plastic sputum plugs and mucosal necrosis. Descriptive statistics and non-parametric tests were employed to analyse the data. Continuous variables were described as mean±sd or median (interquartile range), while categorical variables were represented as frequencies and percentages. To compare groups, independent samples t-tests were employed for continuous variables, while chi-square tests or Fisher’s exact tests were used for categorical variables to assess differences between groups. A significance level of *P*<0.05 was set to determine statistical significance, with all statistical analyses conducted using SPSS or equivalent statistical software.

### Metagenomic data analysis

#### Quality control and preprocessing of sequencing data

In this study, total DNA was extracted from BALF samples and metagenomic libraries were constructed, with blank controls set throughout the experiment to exclude potential contamination. All negative controls were sequenced alongside the biological samples using paired-end mode, and reads detected in the negative controls were removed after sequencing to eliminate interference from environmental or reagent-derived contamination. To guarantee data quality, a comprehensive preprocessing of the raw sequencing data was conducted. The Trimmomatic tool (version 0.40) [20] was employed for quality control purposes. The specific parameter settings used were ILLUMINACLIP: adapters_path: 2:30:10, SLIDINGWINDOW:4:20 and MINLEN:50, which were designed to effectively remove adapter sequences and low-quality reads. Subsequently, the Bowtie2 tool (version 2.5.4) [21] was employed to eliminate host sequences by aligning reads to the human reference genome GRCh38 (hg38), with parameters set to --very-sensitive to optimize alignment accuracy. The efficacy of the data quality control procedure was assessed using the FastQC tool (version 0.12.0), thereby confirming that the processed data were suitable for subsequent analysis.

#### Species diversity analysis

To evaluate the diversity of species within the samples, we employed a series of indices, including the Shannon, Simpson and species richness indices. These indices provide a comprehensive reflection of the richness and evenness of species present in mild and severe cases. Furthermore, a principal coordinate analysis (PCoA) was conducted based on Bray-Curtis dissimilarity metrics, and a diversity plot was constructed for the visualization of relationships among samples. Two-dimensional PCoA was conducted using cmdscale with *k*=2, and the coordinates of the sample points were extracted. Subsequently, the coordinates were merged with the relevant group information, and a permutation multivariate analysis of variance (PERMANOVA) was conducted, with the results calculated using the adonis2 function to obtain *R*² values and *P*-values. Based on the PCoA results, we employed the ggplot2 package to generate scatter plots, incorporating confidence ellipses while modifying colours and styles as necessary.

#### Taxonomy classification and abundance estimation

For the purpose of species annotation, the Kraken2 tool (version 2.1.3) [22] was employed against the standard NCBI non-redundant nucleotide database (nt database), with parameters set to --confidence 0.2. We employed the Bracken tool (version 2.9) [23] for Bayesian estimation in order to ascertain species abundance and its distribution at the phylum, genus and species levels. To identify significant differentially abundant species between the two groups, we employed the LDA effect size (LEfSe) analysis method. Initially, we filtered significant differentially abundant species through the Kruskal–Wallis test and subsequently analysed their taxonomic convergence with the Wilcoxon test. Finally, we set an LDA value greater than 3 to identify the species between mild and severe cases.

### Analysis of liquid chromatography-MS metabolomics

A metabolomic analysis was conducted using liquid chromatography-mass spectrometry (LC-MS) for the qualitative and quantitative assessment of metabolites [24,25]. The data analysis process entailed the application of principal component analysis(PCA) and multivariate analysis(MVA) for the purpose of identifying significantly different metabolites. The specific screening procedure was as follows: initially, significant metabolites were filtered using t-tests (*P*≤0.05) and the VIP values of the first principal component in the orthogonal partial least squares discriminant analysis(OPLS-DA) model [variable importance in projection (VIP) ≥1], while requiring the log fold change to be ≥0. Subsequently, all significant metabolites were ranked by VIP values, selecting the top 20 metabolites deemed most critical in the model. Of these 20 metabolites, those most pertinent to KEGG pathway enrichment analysis were subjected to further investigation. Subsequently, a KEGG pathway enrichment analysis was conducted, resulting in the generation of a rectangular tree diagram. This diagram illustrates the key KEGG pathways [26] associated with the selected metabolites, thereby visualizing their involvement in various biological processes.

### Correlation analysis of multi-factors

In order to investigate the relationships between differentially abundant species, metabolites and clinical indexes in cases of varying severity, a series of correlation analyses was conducted, and a correlation heatmap was generated using the pheatmap package in R (version 4.4.1). The Spearman correlation coefficients (*r* values) between the differentially abundant species identified by LEfSe and the top 20 differentially abundant metabolites ranked by VIP values were calculated in order to assess the degree of correlation. Furthermore, the Spearman correlation coefficients between the differentially abundant species and the clinical indexes were calculated. The heatmap generated using the ComplexHeatmap package provides a clear visual representation of the correlation strength and significance among the variables.

### Network construction of differentially abundant species and metabolites

A correlation network was constructed between differentially abundant species and metabolites using the igraph package in R (version 4.4.1), with Spearman correlation coefficients. Subsequently, the interaction network between species and metabolites was visualized using Gephi software (version 0.10.1), thereby providing an intuitive representation of their interrelations.

### Genotyping and antibiotic resistance profiling of *MP* in clinical isolates

Based on metagenomic sequencing data, we identified the genotyping and macrolide resistance variations of *MP*. After quality control of the raw data, high-quality reads were aligned to the *MP* M129 reference genome (GenBank accession: NC_000912.1) using Snippy software (version 4.6.0) for variant detection. To ensure accuracy, high-confidence SNPs were strictly filtered with the following criteria: sequencing depth (DP) ≥10×, base quality (QUAL) ≥30 and mutant allele frequency ≥0.9. Genotyping was performed based on characteristic mutations: samples with simultaneous T>C mutations at positions 184,991 and 650,584 of the reference genome were identified as P1-2 strains, while those without these mutations (consistent with the reference strain) were identified as P1-1 strains. On this basis, we further identified macrolide resistance variations in the samples. It was observed that the genomic sequences among *MP* strains are highly conserved. All strains exhibited uniformity in utilizing the 23S rRNA fragment of the reference strain M129 as the reference genome sequence. The specific workflow is outlined below: Initially, the BWA software (version 0.7.17) [27] was employed to align clean data with the reference genome, resulting in a SAM file post-alignment. Subsequently, SAMtools software (version 1.17) [28] was employed for the conversion of the SAM file format and the sorting of its contents, resulting in a compressed BAM file. The MarkDuplicates module within the GATK software package (version 3.8) was employed to remove PCR duplicate sequences from the BAM file, resulting in a deduplicated BAM file for further analysis. The HaplotypeCaller module from the GATK software package (version 3.8) was employed to identify SNPs occurring in the genomic sequences, generating a genotype-variance format file for individual strains. Ultimately, the CombineGVCFs and GenotypeGVCFs modules from the GATK software package were employed to merge the gvcf files, resulting in the final variant output VCF file. The resistance mutations in the samples were analysed based on the reported resistance mutation sites in the literature [29,30].

### Establishment of a machine learning model for MPP severity

In this study, we organized the data based on the classification of ‘mild’ and ‘severe’ MPP cases as assessed on the seventh day of hospitalization, dividing the dataset into a training set (Discovery) and a testing set (Validation). The dataset was randomly partitioned into training, testing and independent validation sets (2:2:1), followed by feature selection and model construction using a random forest algorithm applied separately to the metagenomic and metabolomic data. Using the randomForest package, we constructed a random forest model comprising 1,000 decision trees and systematically analysed the importance of each feature. Cross-validation (rfcv) was utilized to determine the optimal number of predictive features, and model performance across different feature quantities was compared through multiple rounds of cross-validation. Based on the top 10 important features identified, we retrained the model using the train function from the caret package and validated it on an external public dataset containing 46 cases of mild and severe *MP* infections (Table S1, available in the online Supplementary Material). The classification performance of the model was comprehensively evaluated using receiver operating characteristic curve analysis.

## Result

### Demographic data and clinical features

On the day of admission, 51 of the 153 enrolled patients met the established clinical diagnostic guidelines for SMPP and were classified accordingly. The remaining 102 patients were initially categorized as mild cases. However, during the second clinical severity assessment on the seventh day of hospitalization, 25 of the initially mild cases showed disease progression within the first week and were subsequently reclassified into the SMPP group. Among them, 10 developed dyspnoea, 4 reported chest pain, 1 presented with thrombocytopenia, 16 exhibited hepatic dysfunction, 14 showed mucocutaneous involvement, 24 showed progression of pulmonary consolidation involving ≥2/3 of a lobe and 6 developed newly affected lobes (Table 1). The results showed that the standard clinical assessment at initial admission achieved an identification accuracy of 67.1% (51/76) for SMPP cases, with nearly one-third of severe cases not being detected in the early disease stage. Severe cases exhibited significant differences from mild cases in clinical manifestations, inflammatory levels, imaging features and prognosis (all *P*<0.05). Specifically, in terms of clinical manifestations, the maximum body temperature of severe cases was significantly higher than that of mild cases on day 7 (*P*<0.001), while dyspnoea (*P*<0.001) and chest pain (*P*=0.028) were observed only in the severe group, and the incidences of hepatic dysfunction and mucocutaneous involvement were also significantly higher in this group (*P*<0.001). Regarding inflammatory levels, the levels of C-reactive protein (CRP), IL-6, lactate dehydrogenase (LDH) and D-dimer in the severe group were significantly higher than those in the mild group (*P*<0.001). In terms of imaging features, the proportion of patients with consolidation area involving ≥2/3 of a lobe was significantly higher in the severe group (*P*<0.001), and the incidences of necrotizing pneumonia, pleural effusion and tree-like casts were also significantly increased (*P*<0.001). Regarding prognosis, the severe group had a significantly longer hospitalization duration (*P*<0.001), a significantly lower proportion of patients with no sequelae (*P*<0.001), and atelectasis and PiBO were observed only in this group (*P*<0.001).

**Table 1.** Clinical features of 153 Chinese children with MPP stratified by disease severity

	At admission			At hospital day 7		
Variable	Severe case (*n*=51)	Mild case (*n*=102)	*P* value	Severe case (*n*=76)	Mild case (*n*=77)	*P* value
Age (years)	6.7±2.3	6.7±2.5	0.851	6.8±2.3	6.6±2.5	0.576
Sex, male/female	31/20	63/39	0.475	50/26	44/33	0.272
**Clinical manifestations**						
Fever, *n* (%)	51 (100)	102 (100)	/	76 (100)	77 (100)	/
Maximum body temperature (Tmax) (°C)	40.0 (39.7, 40.5)	39.0 (39.4, 39.8)	<0.001	40.0 (39.5, 40.3)	39.3 (38.9, 39.6)	<0.001
Cough, *n* (%)	51 (100)	102 (100)	/	76 (100)	77 (100)	/
Wheeze, *n* (%)	2 (4.0)	3 (3.0)	0.748	4 (5.3)	3 (3.9)	0.686
Dyspnoea, *n* (%)	22 (43.1)	0 (0)	<0.001	32 (42.1)	0 (0)	<0.001
Chest pain, *n* (%)	4 (7.8)	0 (0)	0.004	7 (9.2)	0 (0)	0.028
**Extrapulmonary complications, *n* (%)**						
Thrombocytopenia	2 (4.0)	0 (0)	0.044	3 (4.0)	0 (0)	0.078
Hepatic dysfunction	11 (21.2)	0 (0)	<0.001	27 (35.5)	0 (0)	<0.001
Mucocutaneous involvement	15 (29.4)	0 (0)	<0.001	21 (27.6)	0 (0)	<0.001
**Laboratory parameters**						
White blood cell count (WBC) (×10^9^ l^−1^)	7.8 (6.2, 9.8)	7.4 (6.0, 9.3)	0.255	8.1 (6.3, 11.2)	7.3 (5.8, 8.5)	0.023
Neutrophil percentage (%)	74.9±9.8	62.9±12.9	<0.001	73.9±10.3	60.0±12.3	<0.001
Haemoglobin (Hb) (g l^−1^)	116.5±24.4	125.9±11.6	0.010	120.8±10.2	127.5±11.0	<0.001
Platelet count (PLT) (×10^9^ l^−1^)	284.0 (210.0, 340.0)	286.0 (230.0, 346.0)	0.433	289.0 (209.5, 339.8)	280.0 (231.0, 346.0)	0.342
CRP (mg l^−1^)	37.33 (20.1, 87.1)	11.6 (5.6, 21.8)	<0.001	35.4 (14.0, 75.4)	9.5 (4.2, 17.2)	<0.001
Erythrocyte sedimentation rate (ESR) (mm h^−1^)	37.0 (26.3, 51.8)	26.5 (19.0, 39.0)	0.007	33.0 (19.8, 50.8)	28.0 (20.0, 38.0)	0.133
Procalcitonin (PCT) (ng ml^−1^)	0.3 (0.1, 0.5)	0.1 (0.1, 0.2)	<0.001	0.2 (0.1, 0.4)	0.1 (0.1, 0.1)	<0.001
IL-6 (pg ml^−1^)	27.6 (11.0, 71.1)	7.9 (3.2, 20.9)	<0.001	21.9 (8.0, 57.4)	7.2 (3.1, 19.4)	<0.001
LDH (U l^−1^)	553.0 (412.3, 712.5)	303.5 (271.3, 404.0)	<0.001	524.5 (412.2, 701.6)	288.0 (263.0, 335.0)	<0.001
Serum ferritin (SF) (U l^−1^)	259.0 (133.4, 573.1)	86.8 (63.3, 166.9)	<0.001	245.1 (139.4, 558.5)	77.5 (59.7, 97.8)	<0.001
Prothrombin time (PT) (s)	12.1±1.0	11.7±0.8	0.016	12.0±0.9	11.7±0.8	0.028
Activated partial thromboplastin time (APTT) (s)	25.7 (23.3, 28.0)	28.6 (25.5, 31.4)	<0.001	25.7 (23.2, 28.8)	28.9 (26.4, 31.8)	<0.001
Fibrinogen (g l^−1^)	3.8 (3.0, 4.1)	3.4 (3.0, 3.7)	0.022	3.6 (3.0, 4.1)	3.3 (3.0, 3.7)	0.05
D-Dimer (μg ml^−1^)	1.8 (0.9, 3.0)	0.4 (0.4, 0.8)	<0.001	1.6 (0.9, 3.2)	0.4 (0.4, 0.5)	<0.001
**Chest CT imaging, *n* (%)**						
Consolidation area ≥2/3 lobe	51 (100)	0 (0)	<0.001	75 (98.7)	0 (0)	<0.001
Consolidation involvement 2 lobes	34 (66.7)	0 (0)	<0.001	40 (52.6)	0 (0)	<0.001
Necrotizing pneumonia	0 (0)	0 (0)	/	18 (23.7)	1 (1.3)	<0.001
Pleural effusion	20 (39.2)	0 (0)	<0.001	36 (47.4)	4 (5.2)	<0.001
Pulmonary thrombosis	0 (0)	0 (0)	/	9 (11.8)	0 (0)	<0.001
**Bronchoscopy findings, *n* (%)**						
Tree-like casts	32 (62.7)	0 (0)	<0.001	62 (81.6)	0 (0)	<0.001
Mucosal erosion	14 (27.5)	0 (0)	<0.001	30 (39.5)	1 (1.3)	<0.001
**Therapeutic indicator, *n* (%)**						
Hospitalization duration (day)	/	/	/	13 (10, 15)	7 (8, 9)	<0.001
No sequelae	/	/	/	28 (36.8)	77 (100)	<0.001
Atelectasis	/	/	/	19 (25.0)	0 (0)	<0.001
Post-infectious bronchiolitis obliterans (PiBO)	/	/	/	10 (13.2)	0 (0)	<0.001
Lost to follow-up	/	/	/	19 (25.0)	0 (0)	<0.001

### Sample collection and data organization

A total of 153 enrolled patients were included in this study. BALF samples were collected from all patients on the second day of hospitalization and subsequently subjected to metagenomic and untargeted metabolomic sequencing, generating over 1 TB of raw data. Following quality control and preprocessing, high levels of host contamination (>90%) were observed in the BALF samples. Nevertheless, more than 110 GB of effective data across all samples were retained for downstream analysis. The median raw sequencing read count was high, but the median retained microbial read count dropped markedly after host depletion (Fig. S1), yielding a host removal rate of over 70%. Among these, metagenomic analysis was performed on 152 samples (one sample was discarded due to poor sequencing data quality), while untargeted metabolomic analysis included all 153 samples, with no samples excluded.

### Comparative analysis of the metagenomic profiles between MMPP and SMPP

**Fig. 1.**
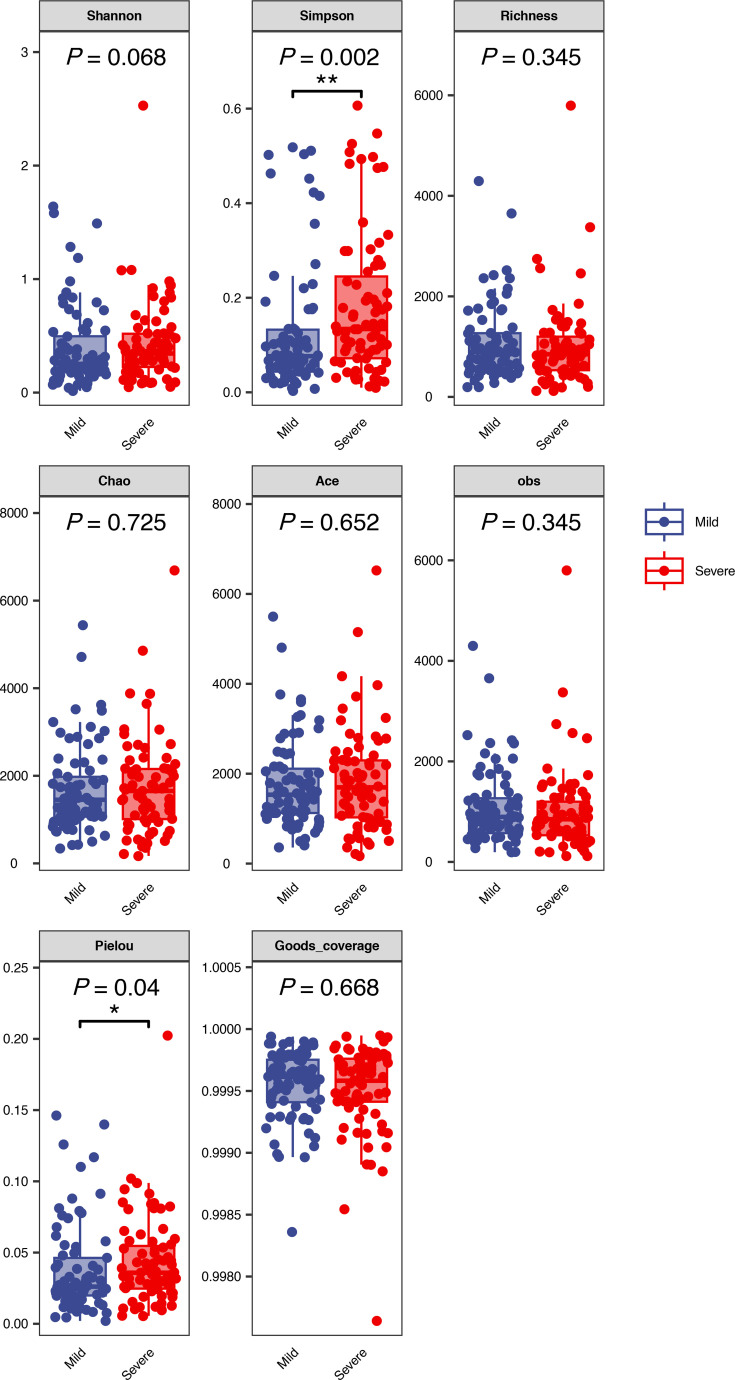
*α*-Diversity analysis of mild and severe *MP* infections.

**Fig. 2.**
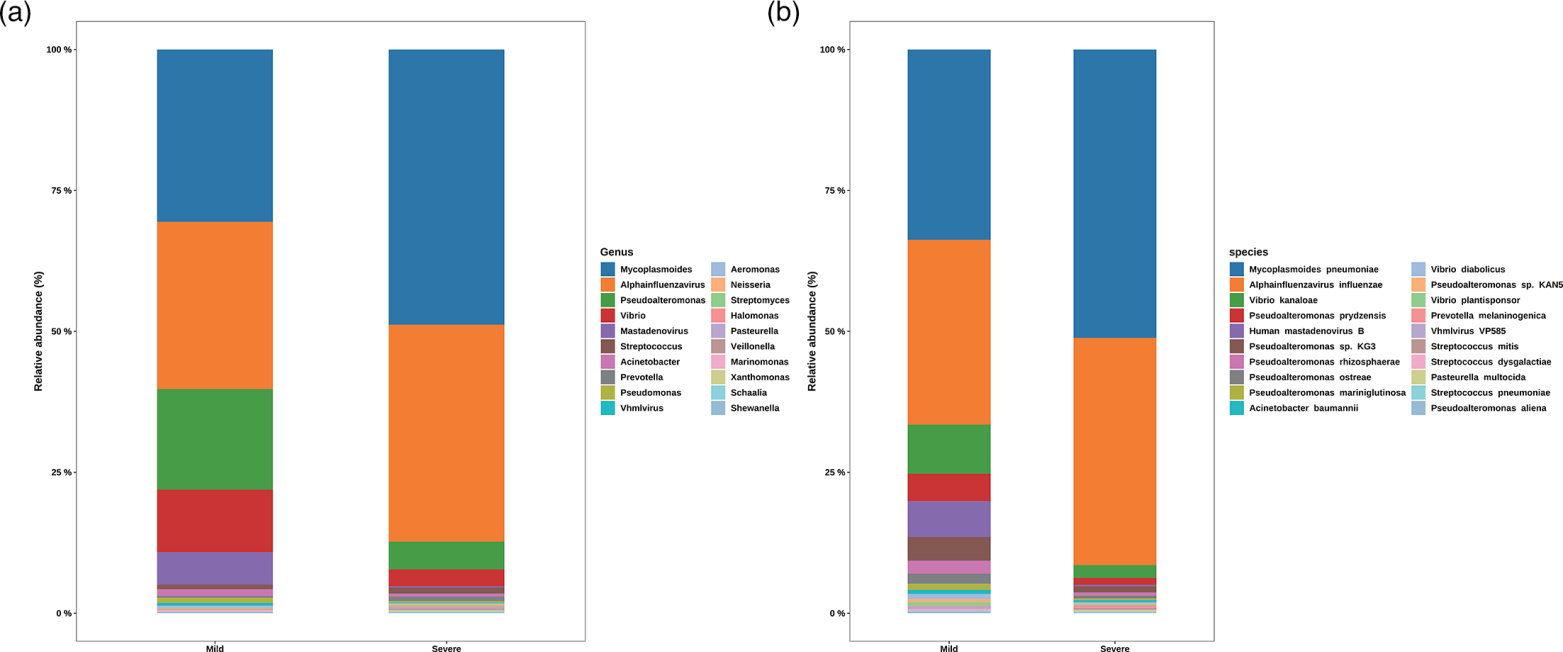
Analysis of relative abundance of microbial community composition in mild and severe *MP* patients. (**a**) Genus level. (b) Species level.

**Fig. 3.**
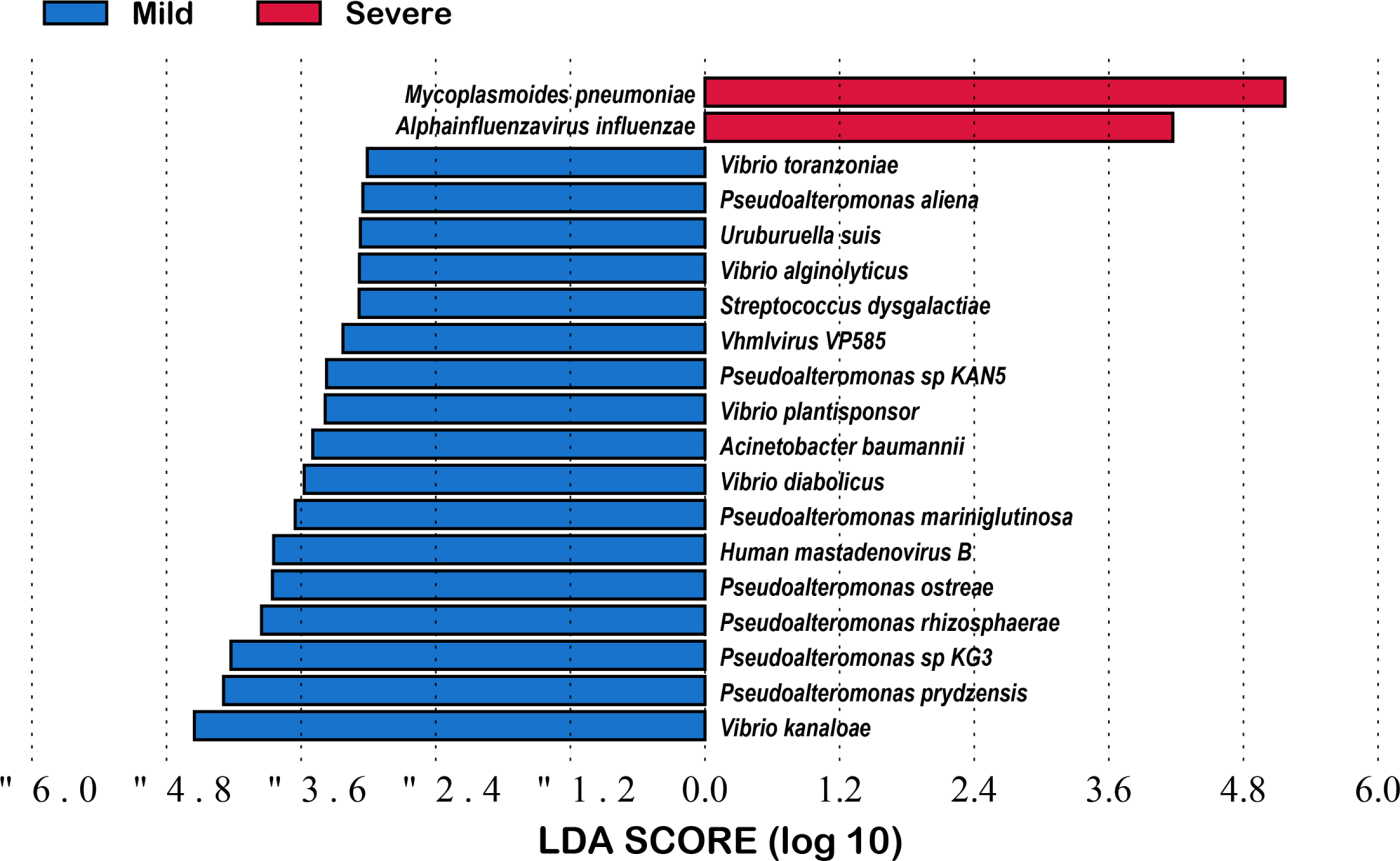
LDA analysis of species abundance and classification features in mild and severe *MP* patients.

A comparative analysis of the microbial diversity of MMPP and SMPP was conducted based on metagenomic data derived from BALF. To assess alpha diversity, six commonly used indices were selected, including the Shannon, Simpson, Chao indices, etc. (Fig. 1). With the exception of the Simpson and Pielou indices, no significant differences were observed in the remaining diversity indices between the mild and severe cases. The Simpson index showed a significantly higher diversity in the severe group compared to the mild group, and the Pielou index exhibited a comparable pattern (*P*≤0.05). The PCoA results indicated that the differences between the mild and severe cases were not significant, with the majority of samples from both groups exhibiting substantial overlap (Fig. S2). At the phylum level (Fig. S3), the species identified in both the mild and severe groups were predominantly affiliated with the *Mycoplasmatota*, *Negarnaviricota*, *Pseudomonadota* and *Preplasmiviricota*. In comparison to the mild group, the proportion of *Mycoplasmatota* and *Negarnaviricota* increased in the severe group, while the proportion of *Pseudomonadota* and *Preplasmiviricota* decreased. At the genus level (Fig. 2a), the dominant species were *Mycoplasmoides*, *Alphainfluenzavirus*, *Pseudoalteromonas* and *Vibrio*. As observed at the phylum level, the proportion of *Mycoplasmoides* and *Alphainfluenzavirus* increased in the severe group, while that of other dominant species decreased. At the species level (Fig. 2b), the composition of the dominant species was found to be consistent between the mild and severe groups, although their proportions differed. In the severe group, *MP* and *A. influenzae* constituted over 80% of the total. This figure was considerably higher than the proportion of the two species in the mild group. Furthermore, the relative abundance of other dominant species, such as *Vibrio kanaloae* and *Pseudoalteromonas prydzensis*, was diminished in the severe group. Similarly, differential abundance species identification yielded comparable results, with *MP* and *A. influenzae* remaining significantly enriched in the severe group. The mild group exhibited a significantly higher number of differential abundance species, predominantly belonging to the genera *Vibrio* and *Pseudoalteromonas.* The most notable differences were observed in *V. kanaloae* and *P. prydzensis* (LDA >4) (Fig. 3). Based on the analysis of 152 samples, we found that all P1-1 type samples (137 cases, 90.1%) were double-negative at both loci, while all P1-2 type samples (15 cases, 9.9%) carried at least one positive locus. Among the P1-2 type, double-positive cases accounted for 66.7% (10/15). Our results demonstrate that P1 typing effectively distinguishes the two strain types, with the double-positive subtype being predominant in P1-2 (Table S2). It is also noteworthy that the analysis included an investigation of macrolide resistance based on 23S rRNA mutations in *MP* using metagenomic data. The study revealed that the macrolide resistance rate exceeded 70% in both the mild and severe cases, with the resistance rate in the severe group exceeding 85% and over 60% in the mild group. It is noteworthy that the macrolide resistance mutations observed in both groups originated from the A2063G site of the 23S rRNA (Table 2).

**Table 2.** Detection of macrolide-resistant strains of bacteria based on 23S rRNA mutations

Type	No. of macrolide-resistant strains	A2063G	A2063T	A2064G	A2064C	Drug resistance rate (%)
MMPP	66	66	0	0	0	86.84
SMPP	47	47	0	0	0	61.84
Total	113	113	0	0	0	74.34

### Comparative analysis of non-targeted metabolomics between mild and severe cases with MPP

**Fig. 4.**
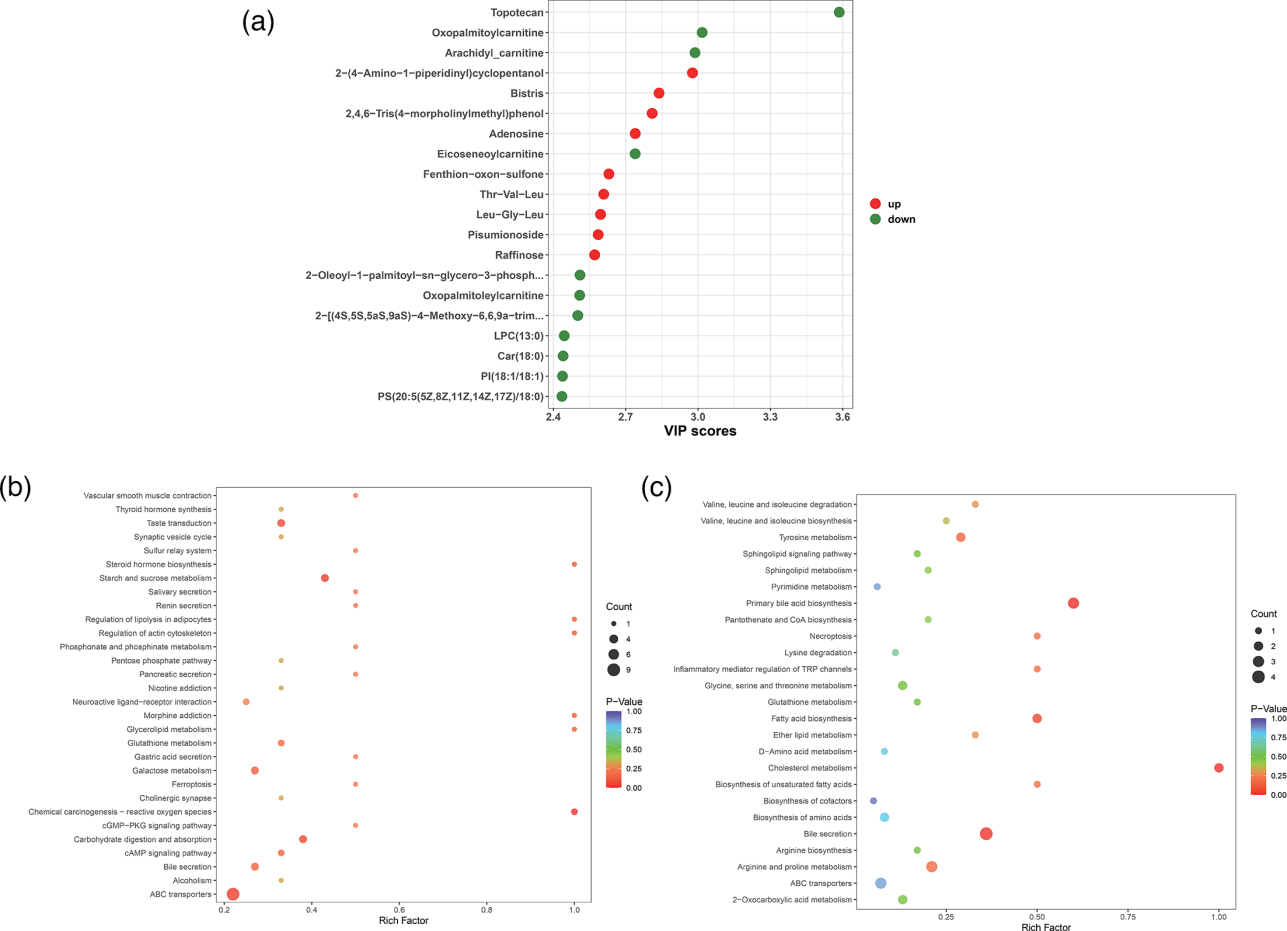
Analysis of differential metabolites between mild and severe *MP* groups. (**a**) Differential metabolites between mild and severe. (**b**) Metabolites enriched in mild group pathways. (**c**) Metabolites enriched in severe group pathways.

Metabolomic data from BALF, detecting over 1,000 metabolites, were used to compare the metabolite profiles between mild and severe cases with MPP. The OPLS-DA analysis revealed significant differences in metabolite levels between mild and severe cases, with samples within the same group exhibiting relatively high clustering (Fig. S4). Subsequently, the differential metabolites between the mild and severe groups were compared. Based on the quantitative metabolomic results, differential metabolites were also identified using VIP values, with the top 20 with VIP >1 selected for presentation (Fig. 4a). The results demonstrated that topotecan was significantly enriched in the severe group (VIP >3), while oxopalmitoylcarnitine and arachidyl-carnitine also exhibited notable differences in the severe group. Conversely, multiple metabolites, including 2-(4-amino-1-piperidinyl)cyclopentanol and Bistris, were upregulated in the mild group. The functional enrichment of the differential metabolites revealed that those enriched in the mild group were significantly involved in pathways such as ABC transporters, starch and sucrose metabolism and carbohydrate digestion and absorption (Fig. 4b). In the severe group, the enriched metabolites primarily participated in pathways such as bile secretion and primary bile acid biosynthesis, although this was not a significant finding (Fig. 4c).

### Correlation analysis of mild and severe cases with MPP

**Fig. 5.**
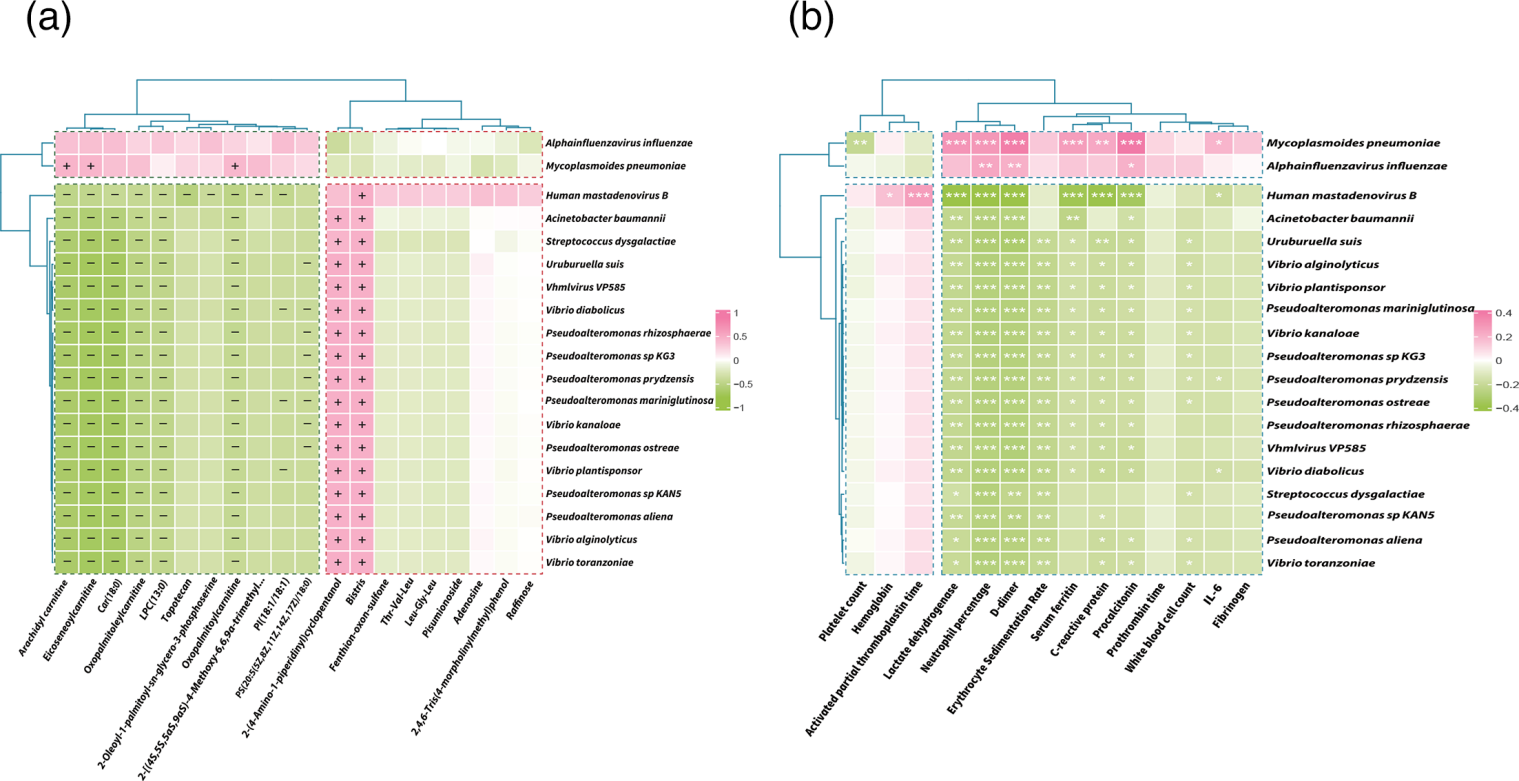
Heatmap study of the correlation between species, metabolites and clinical features in mild and severe *MP* patients. (**a**) Metabolites vs. microbiota. (**b**) Clinical indexes vs. microbiota.

The study aimed to analyse correlations among micro-organisms, metabolites and clinical indicators in MPP cases to elucidate interactions between the lung microbiota and the host. Firstly, a correlation network was constructed between differential micro-organisms and metabolites in MMPP and SMPP cases (Fig. S5). The majority of nodes in the network exhibited relatively uniform degrees of connectivity. It is noteworthy that *MP* was linked to various metabolites, including topotecan and oxopalmitoylcarnitine, which were significantly enriched in the severe group, as well as 2-(4-amino-1-piperidinyl)cyclopentanol and Bistris, which were significantly enriched in the mild group. Similar observations were made with regard to other micro-organisms, including *A. influenzae*, which was enriched in the severe group, and *V. kanaloae*, which was enriched in the mild group. The correlation heatmap revealed that the two species enriched in the severe group (*MP* and *A. influenzae*) exhibited weak correlations with the majority of differential metabolites (Fig. 5a). Only *MP* demonstrated positive correlations with a limited number of metabolites, including arachidyl carnitine and eicosenoic acid carnitine. It is noteworthy that the micro-organisms that were enriched in the mild group exhibited a significant correlation with a considerable number of metabolites. These metabolites were found to be universally associated with all the micro-organisms in the mild group. For instance, arachidyl carnitine and eicosenoic carnitine exhibited notable negative correlations with the micro-organisms in the mild group, whereas Bistris demonstrated significant positive correlations. Subsequently, the correlations between the abundance of micro-organisms and clinical indicators were analysed, and it was found that two micro-organisms in the severe group were positively correlated with several clinical indicators related to inflammation (e.g. CRP, procalcitonin and LDH), with *MP* showing stronger positive correlations. However, the majority of species in the mild group exhibited a negative correlation with the majority of clinical indicators, with the exception of *Human mastadenovirus B*, which demonstrated a robust and statistically significant positive correlation with activated partial thromboplastin time (Fig. 5b).

### Establishment of a multi-factor prediction model for mild and severe cases with MPP

**Fig. 6.**
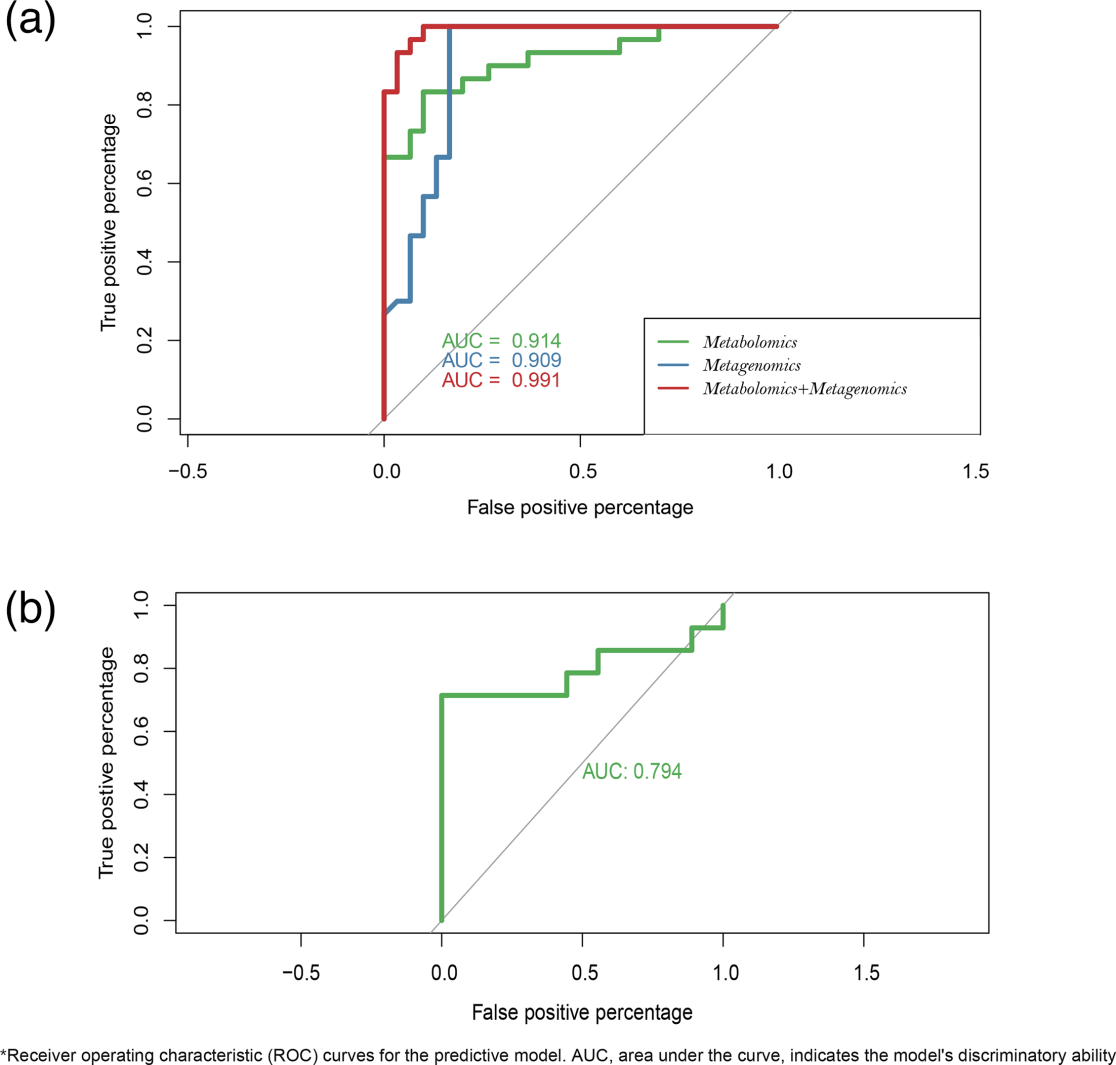
Establishment of a machine learning model for mild and severe *MP* patients. (**a**) Multi-factor model in the study datasets. (**b**) Multi-factor model in the study datasets and extra public datasets.

This study developed an early warning model for distinguishing between MMPP and SMPP based on metagenomic and metabolomic data from BALF samples. A phased modelling strategy was employed: initial models using single data types achieved area under the curve (AUC) values of 0.909 for metagenomics and 0.914 for metabolomics. Integration of multi-omics data significantly enhanced predictive performance (AUC=0.991) (Fig. 6a). External validation using publicly available metagenomic data from MPP cases demonstrated maintained discriminative ability (AUC=0.794), confirming the model’s robustness and potential clinical applicability (Fig. 6b).

## Discussion

MPP is a prevalent respiratory disease, with a particularly high incidence among children. A substantial body of research has been conducted on *MP* infection, employing a range of techniques, including microbial culture, PCR and next-generation sequencing. In comparison to alternative detection methodologies, next-generation sequencing offers a number of advantages, including high accuracy and the capacity for *in situ* detection. In particular, metagenomic detection based on next-generation sequencing provides a solution to the difficulties associated with culturing *MP* and offers an improvement in the accuracy of PCR methods. A number of studies have identified the potential applications of metagenomic detection in the context of lung infections. There are notable clinical differences between mild and severe lung infections caused by *MP*, yet effective methods for distinguishing between them remain elusive. What is more, there has been a paucity of research investigating the mechanisms of micro-organism–host interactions and the development of corresponding machine learning models. In this study, we conducted an investigation of MMPP and SMPP, including micro-organisms and metabolites, for the first time. This allowed us to identify significant differences in microbial communities and metabolites, as well as their correlations, with the aim of gaining a deeper understanding of micro-organism–host interaction mechanisms. This study systematically evaluated the predictive efficacy of different omics data. The results demonstrated that prediction models based solely on metagenomic data (AUC=0.909) or metabolomic data (AUC=0.914) significantly outperformed the standard clinical assessment at initial admission (identification accuracy: 67.1%). Integration of these two omics data types further improved model performance (AUC=0.991), with external validation confirming maintained robust discriminative ability (AUC=0.794). These findings indicate that a multi-omics integration strategy can effectively overcome the limitations of traditional clinical evaluation, and the combined analysis of metagenomic and metabolomic data provides crucial technical support for early and precise diagnosis of MPP. However, although the metagenomic signatures demonstrated robustness, the generalizability of the full integrated model (including metabolomic features) remains to be validated pending the availability of public multi-omics datasets.

With regard to microbiomics, no significant differences in microbial diversity were observed between MMPP and SMPP cases, thereby indicating that the clinical differences between these two categories of cases are primarily due to changes in a small subset of bacterial communities. Subsequent analyses of species composition and abundance differences corroborated this finding. It was observed that at the phylum, genus and species levels, the abundance of *Mycoplasma* communities was significantly higher in the severe group. It is noteworthy that, in addition to the significantly increased abundance of *MP*, *A. influenzae* also had a prominent presence in the severe group. Its proportion and degree of difference in the severe group were second only to *MP*, indicating that the cause of SMPP may not be solely attributable to *MP*. Additionally, this suggests that co-infection with *A. influenzae* may be a risk factor for exacerbating the disease. Moreover, the common macrolide resistance sites in *MP* were analysed, with the result that the overall resistance rate exceeded 70% in mild and severe cases (exceeding 80% in severe cases) and was due to a single site. The high macrolide resistance also indicates the necessity for meticulous antibiotic selection in future treatments. An investigation was conducted into the differences in metabolites between MMPP and SMPP cases. The results demonstrated that topotecan was significantly more prevalent in the severe group. It is a recently developed cytotoxic agent that acts as a topoisomerase I inhibitor, an enzyme that is essential for DNA replication [31]. It forms stable covalent complexes with the DNA/topoisomerase I aggregate, resulting in the fragmentation of DNA strands and the subsequent induction of apoptosis and cell death. Furthermore, significant differences were observed in the levels of oxopalmitoylcarnitine and arachidyl carnitine in the severe group. These compounds are intimately associated with the metabolic processes occurring within the human body. Oxopalmitoylcarnitine is a compound belonging to the class of compounds known as carnitine derivatives [32]. It is primarily responsible for the transport of long-chain fatty acids into the mitochondria, where they are oxidized to produce energy. In the context of biomedical research, oxopalmitoylcarnitine may be associated with a range of health issues, including metabolic disorders and cardiovascular diseases. Arachidyl carnitine plays a pivotal role in maintaining the equilibrium of intracellular glucose and lipid metabolism [33].

In this study, we employed a multi-omics approach integrating microbiomic, metabolomic and clinical data to elucidate the underlying mechanisms of MPP. Firstly, a correlation network was constructed between the micro-organisms and metabolites. The network and correlation heatmap revealed that *MP* and *A. influenzae* were linked to multiple metabolites that were significantly enriched in the severe group (e.g. topotecan, oxopalmitoylcarnitine and arachidyl carnitine). This suggests that changes in host cell toxicity and energy metabolism are closely related to micro-organisms. It is noteworthy that, with the exception of *H. mastadenovirus B*, the majority of micro-organisms that were significantly enriched in the mild group were negatively correlated with the aforementioned metabolites. This may indicate that these microbial communities do not exacerbate MPP. At the level of associations between micro-organisms and clinical indicators, a similar pattern was observed with regard to the correlations with immune markers, whereby most micro-organisms enriched in the mild group were negatively correlated with these. Conversely, *MP* and *A. influenzae* were found to be positively correlated with the majority of immune indicators, indicating that these species are capable of activating the host’s immune response.

It should be noted that our study is not without limitations. The sample size is insufficient for the purposes of this study. Despite the extensive research conducted on *MP*, there is a paucity of studies investigating the differences between mild and severe cases, as well as the associated data. In subsequent studies, we will increase the sample size in order to confirm the generalizability of the current conclusions. Secondly, high-quality genomes were not obtained from *MP* samples. The high levels of host contamination observed in the lung metagenome present a significant challenge in obtaining high-quality *MP* genomic sequences based on metagenomic binning results. Ultimately, further functional experiments are required to elucidate the underlying mechanisms involving the relevant key metabolites in greater detail.

## Conclusion

Through the analysis of BALF samples from 153 children with MPP, this study found that the severe group had a longer hospitalization time compared to the mild group, with significant differences in clinical manifestations and biochemical indicators. Moreover, the proportions of consolidation, pulmonary necrosis, pleural effusion, pulmonary embolism, plastic sputum plugs and bronchial mucosal necrosis were significantly higher in the severe group. Metagenomic sequencing showed higher proportions of *MP* and *A. influenzae* in severe cases. Metabolomic analysis revealed significant enrichment of specific metabolites in these cases. In addition, the macrolide resistance rate of *MP* exceeded 85% in the severe group, compared to over 60% in the mild group. The multi-factor prediction model, combining metagenomic and metabolomic data, effectively distinguished between MMPP and SMPP, demonstrating high classification performance. We hope our findings will provide a valid basis for the future treatment of mild and severe cases with MPP.

## Supplementary material

10.1099/mgen.0.001717 Uncited Supplementary Material 1.

10.1099/mgen.0.001717 Uncited Supplementary Material 2.

## Abbreviations

AUC: area under the curve
BALF: bronchoalveolar lavage fluid
CRP: C-reactive protein
CT: computed tomography
LDH: lactate dehydrogenase
LEfSe: LDA effect size
MMPP: mild *Mycoplasma pneumoniae* pneumonia
*MP*: *Mycoplasma pneumoniae*
MPP: *Mycoplasma pneumoniae* pneumonia
PCoA: principal coordinate analysis
PiBO: post-infectious bronchiolitis obliterans
SMPP: severe *Mycoplasma pneumoniae* pneumonia
VIP: variable importance in projection
