# Supplementary material for: Unveiling metagenomic and metabolomic signatures in mild and severe pneumonia caused by Mycoplasma pneumoniae in children
Source: Microb Genom. 2026 May 20;12(5):001717. doi: 10.1099/mgen.0.001717 (PMC13189360; doi:10.1099/mgen.0.001717)
Supplement: Uncited Supplementary Material 1. [file mgen-12-01717-s001.pdf]

Figure S1

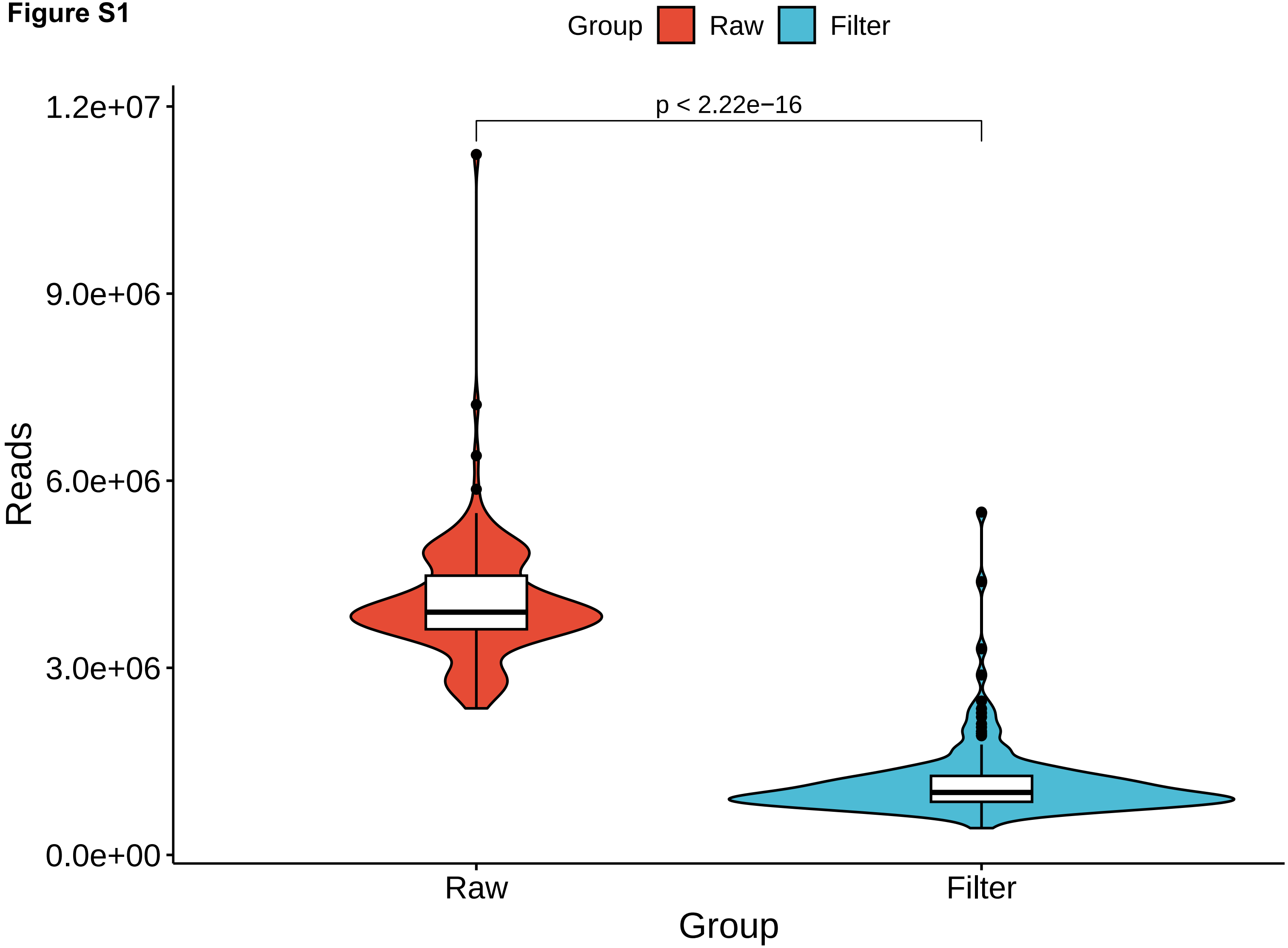

Group
Raw
Filter
p < 2.22e−16
1.2e+07
9.0e+06
6.0e+06
3.0e+06
0.0e+00
Reads
Raw
Filter
Group

Figure S2

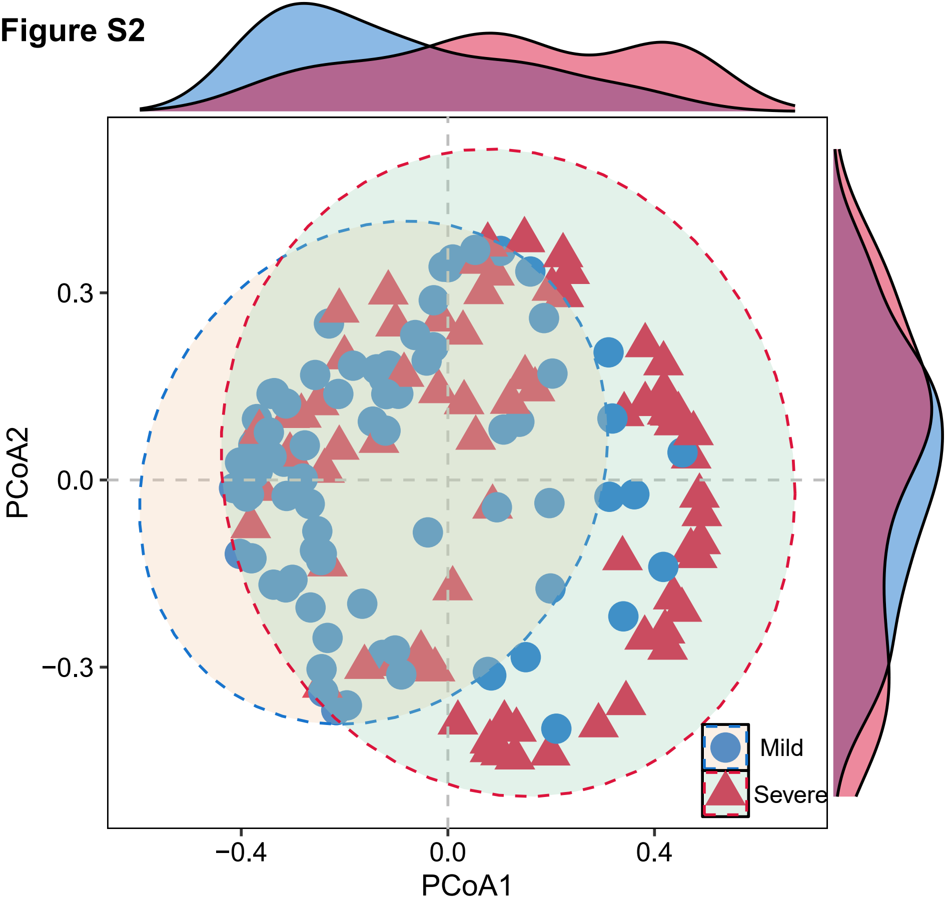

adonisR2: 0.05 P−value: 0.001

Figure S3

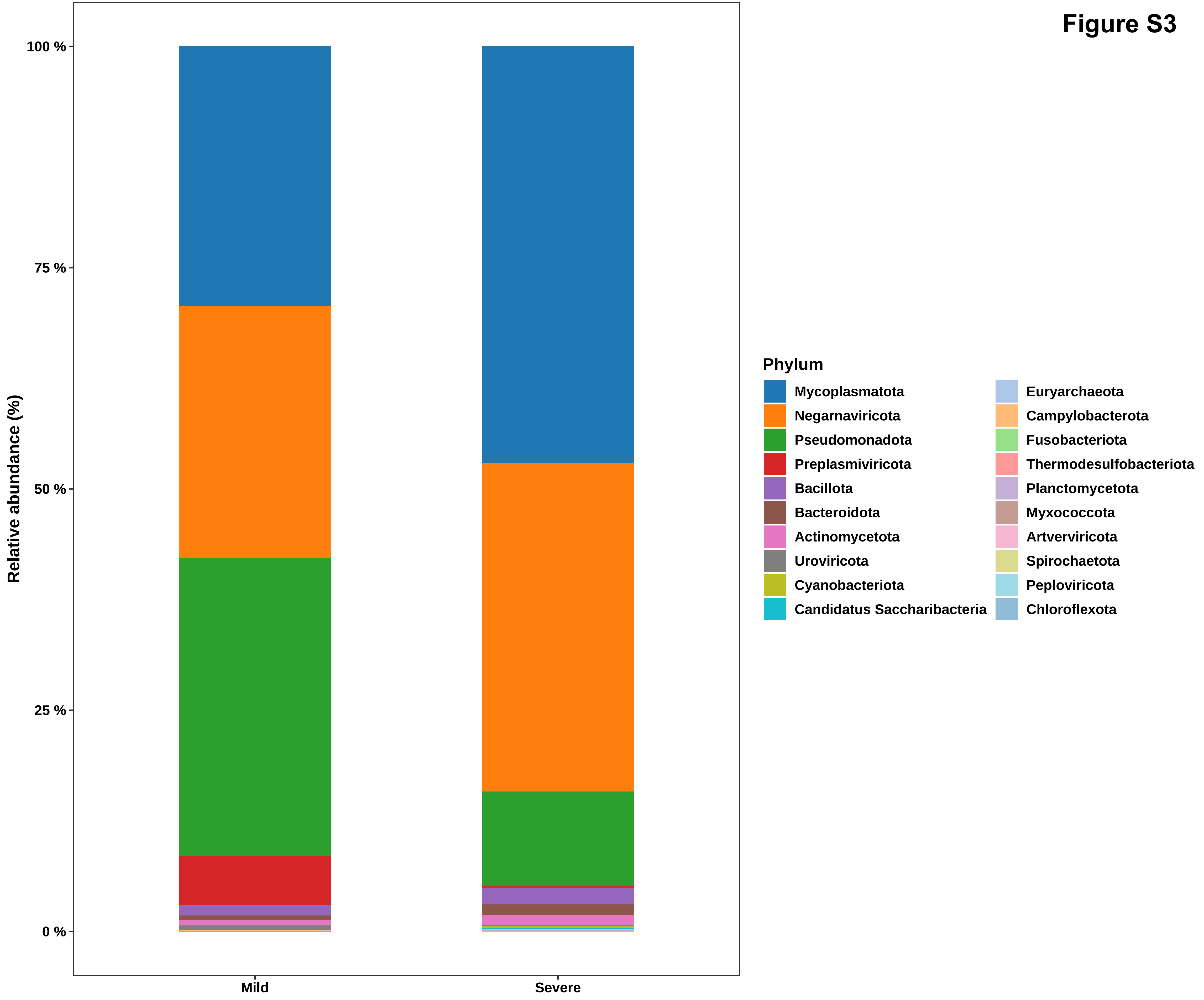

100 %
75 %
50 %
25 %
0 %
Relative abundance (%)
Mild
Severe
Phylum
Mycoplasmatota
Negarnaviricota
Pseudomonadota
Preplasmiviricota
Bacillota
Bacteroidota
Actinomycetota
Uroviricota
Cyanobacteriota
Candidatus Saccharibacteria
Euryarchaeota
Campylobacterota
Fusobacteriota
Thermodesulfobacteriota
Planctomycetota
Myxococcota
Artverviricota
Spirochaetota
Peploviricota
Chloroflexota

Figure S4

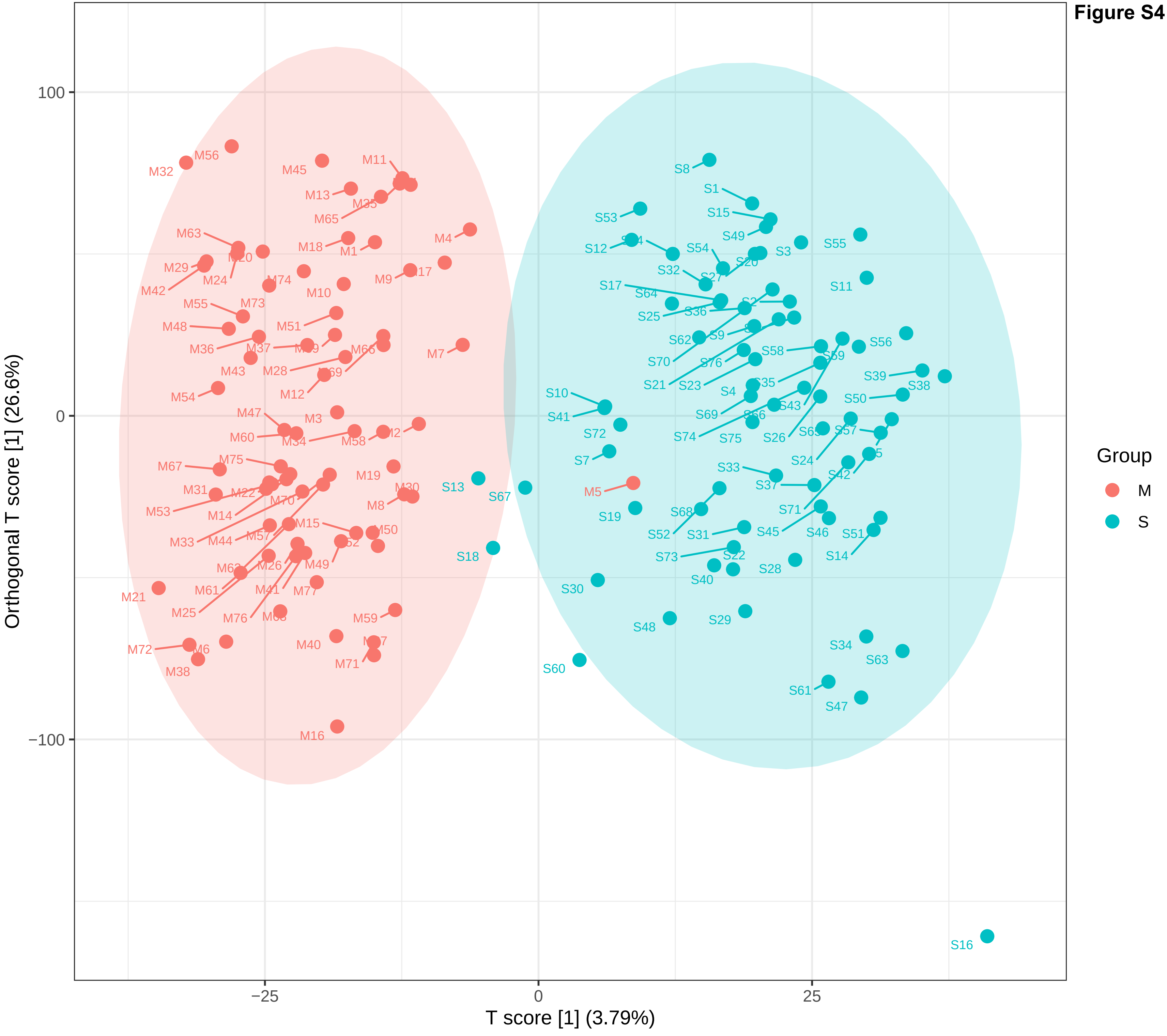

Figure S5

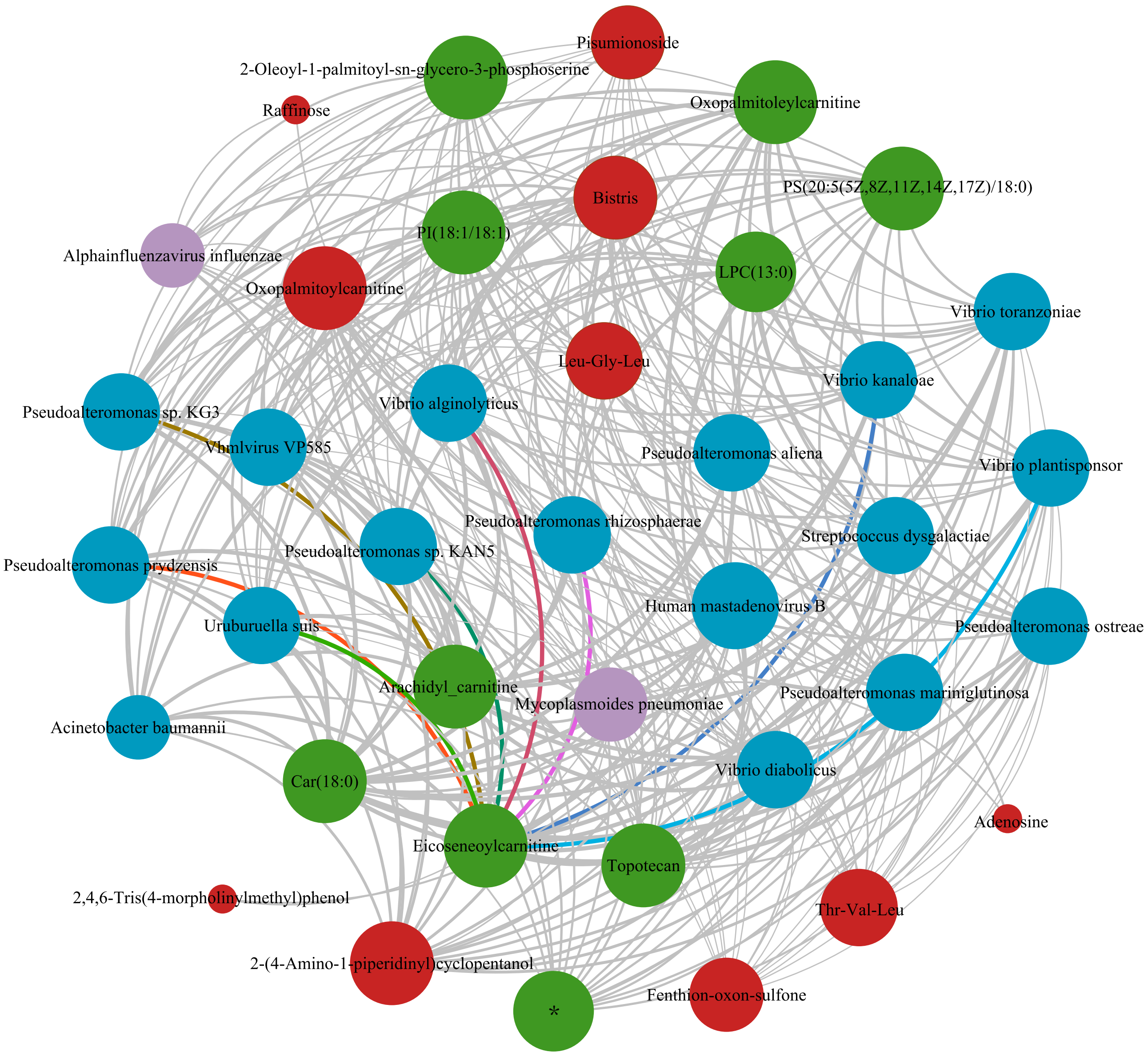

* 2-[(4S,5S,5aS,9aS)-4-Methoxy-6,6,9a-trimethyl-5-[(2E,4E,6E)-octa-2,4,6-trienoyl]oxy-1-oxo-4,5,5a,7,8,9-hexahydro-3H-benzo[e]isoindol-2-yl]pentanedioic acid
